# Development and Validation of a Clinical and Computerised Decision Support System for Management of Hypertension (DSS-HTN) at a Primary Health Care (PHC) Setting

**DOI:** 10.1371/journal.pone.0079638

**Published:** 2013-11-05

**Authors:** Raghupathy Anchala, Emanuele Di Angelantonio, Dorairaj Prabhakaran, Oscar H. Franco

**Affiliations:** 1 Department of Public Health & Primary Care, University of Cambridge, Cambridge, United Kingdom; 2 Public Health Foundation of India, New Delhi, India; 3 Centre for Chronic Disease Control, New Delhi, India; 4 Department of Epidemiology, Erasmus MC, Rotterdam, The Netherlands; University of New South Wales, Australia

## Abstract

**Background:**

Hypertension remains the top global cause of disease burden. Decision support systems (DSS) could provide an adequate and cost-effective means to improve the management of hypertension at a primary health care (PHC) level in a developing country, nevertheless evidence on this regard is rather limited.

**Methods:**

*Development* *of* *DSS* *software* was based on an algorithmic approach for (a) evaluation of a hypertensive patient, (b) risk stratification (c) drug management and (d) lifestyle interventions, based on Indian guidelines for hypertension II (2007). The *beta* *testing* *of* *DSS* *software* involved a feedback from the end users of the system on the contents of the user interface. *Software* *validation and piloting* was done in field, wherein the virtual recommendations and advice given by the DSS were compared with two independent experts (government doctors from the non-participating PHC centers).

**Results:**

The overall percent agreement between the DSS and independent experts among 60 hypertensives on drug management was 85% (95% CI: 83.61 - 85.25). The kappa statistic for overall agreement for drug management was 0.659 (95% CI: 0.457 - 0.862) indicating a substantial degree of agreement beyond chance at an alpha fixed at 0.05 with 80% power. Receiver operator curve (ROC) showed a good accuracy for the DSS, wherein, the area under curve (AUC) was 0.848 (95% CI: 0.741 - 0.948). Sensitivity and specificity of the DSS were 83.33 and 85.71% respectively when compared with independent experts.

**Conclusion:**

A point of care, pilot tested and validated DSS for management of hypertension has been developed in a resource constrained low and middle income setting and could contribute to improved management of hypertension at a primary health care level.

## Introduction

Hypertension exerts a substantial public health burden on cardiovascular health status and health care systems in India [1,2]. The pooled prevalence of hypertension is estimated to be 25% (95% CI: 11.66 - 44.8 in males and 13.68 - 44.5 in females) and 10% (95% CI: 3.7 - 24 in males and 3.69 - 17 in females) in urban and rural areas of India respectively [3-9].. By 2025, the rate of hypertension (in %) has been projected to go up to around 22.9 and 23.6 from the existing rates of 20.6 and 20.9 (in 2000) for Indian males and females respectively [6]. However, only one fourth of Indian patients on anti-hypertensives achieve blood pressure control [7]. Recent studies have shown that physician adherence to evidence based and standardized medical care results in achieving adequate blood pressure control among hypertensive patients [10,11]. Clinical guidelines and algorithms at the point of health care delivery, in the form of decision making aids to the health care providers, such as clinical and or computerised decision support systems (DSS) are a possible way to improve the standard of care delivery, more so, in a resource constrained primary health care setting [11,12]. 

Clinical and computerised decision support systems have been developed, validated and field tested in the western world for management of hypertension during the last decade [13–15]. Mixed results have been shown for DSS in the management of hypertension in the developed world for patient outcomes, but have shown that they may improve the Physician performance [10-12]. An improvement in the quality of antihypertensive treatment, concurrently leading to a considerable reduction in drug costs have been shown for DSS [16]. There exist no studies in a low and middle income country (LMIC), wherein, a clinical decision support system, either computerised or non-computerised, has been shown to aid clinical decision making and in management of hypertension. 

Hence, we performed a study to find out the ease of building a clinical decision support system, its validity, and to assess the utility of DSS in managing hypertension at a primary health care level in a LMIC (India). The primary purpose for developing the DSS software was to help the end user (health care providers-physicians serving at the primary health care level) to (a) undertake a thorough evaluation of risk factors for hypertension and future cardiovascular diseases (b) to classify the risk levels for progression to future cardiovascular diseases (c) to follow a software prompted algorithmic guideline based drug management (which would be developed based on Indian Hypertension guidelines II, 2007 [17] and (d) to give alerts on the counseling on lifestyle changes and adherence to medication. The aim was to develop, pilot test and validate a decision support system for hypertensive patients. Improvement in patient outcomes (reduction in blood pressure and improvement in BP control rates) and physician skills and practitioners performance (uptake of evidence based guidelines for hypertension by the primary care physicians) were the two main issues that we attempted to address by developing and deploying a DSS in PHCs.

## Methods

**Figure 1:**
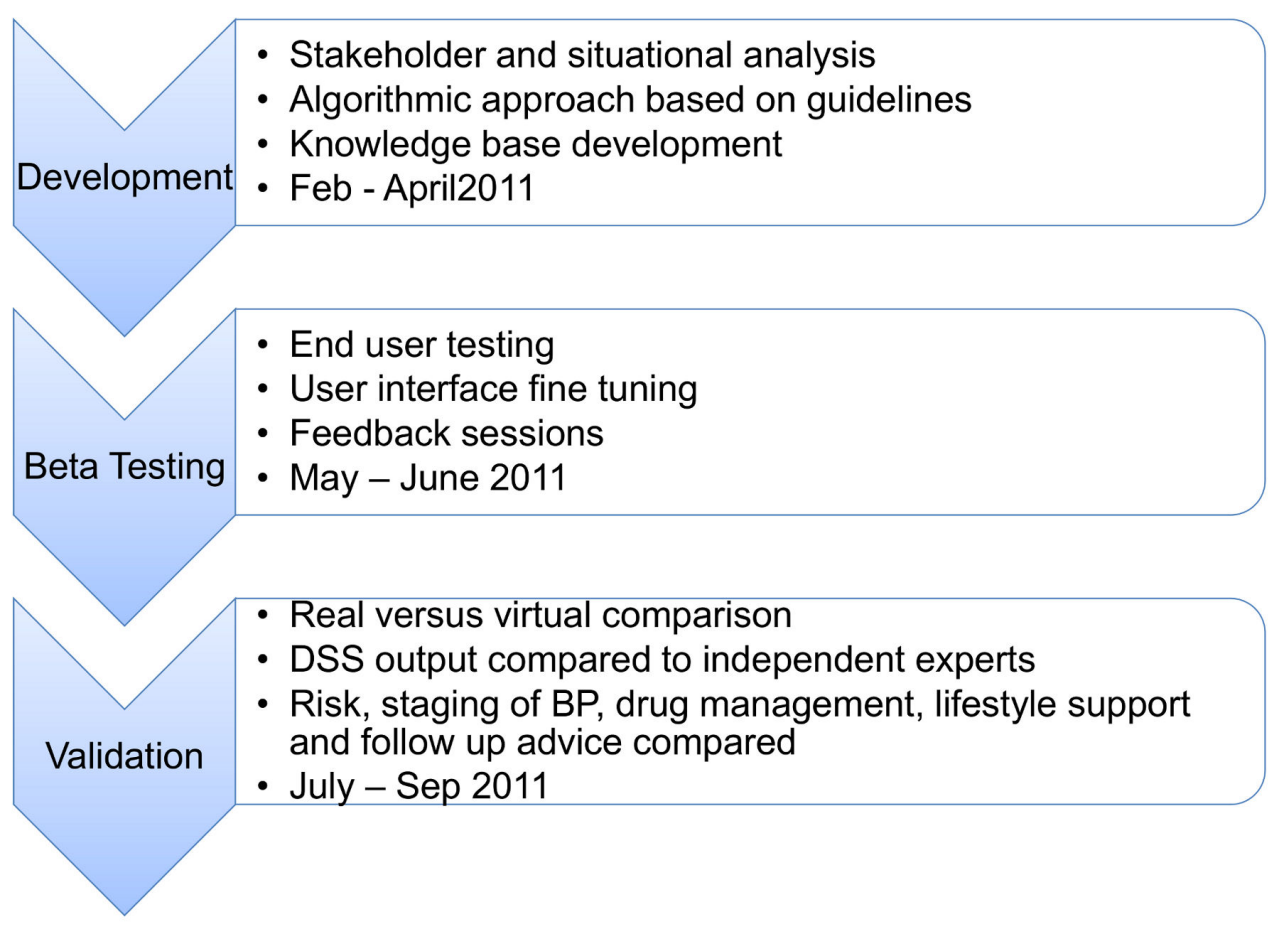
Phases of DSS development and validation.

The phases of DSS development and validation are summarized in Figure **1**.

### Phase I - Development of DSS software

The knowledge base for the evaluation, staging and risk stratification of a hypertensive patient; algorithmic drug based management and lifestyle interventions in the DSS were developed based on the Indian Hypertension II (2007) guidelines, which have been developed by the Association of Physicians of India (API) and endorsed by the Cardiological Society of India, Hypertension Society of India and the API [17]. Stakeholder and situational analysis formed a major part of the development exercise. Focus group discussions and semi structured questionnaires were done with the consenting 34 Primary Health Care (PHC) doctors and nurse assistants in the Mahabubnagar District (least developed district in the state) Andhra Pradesh State, India (34 out of a total 84.PHC doctors from the district consented to be a part of the study) during the months of April - May 2011. Their opinions, on what kind of clinical support would be required at their resource limited settings were elicited. Leading questions on feasibility and operational issues were deliberated at length to find out their usual practice and management of hypertensive patients reporting to their PHCs. All of the participants suggested development of low cost open source health technology platforms with options for quick scalability and easy deployment as the primary requirements for the DSS. Written informed consent was obtained from physicians and nursing assistants willing to voluntarily undertake testing, implementing and validating the DSS software. The study protocol was approved by the Human Biology Ethical Research Committee, University of Cambridge and the Institutional Ethical Committee of the Public Health Foundation of India. 

**Figure 2:**
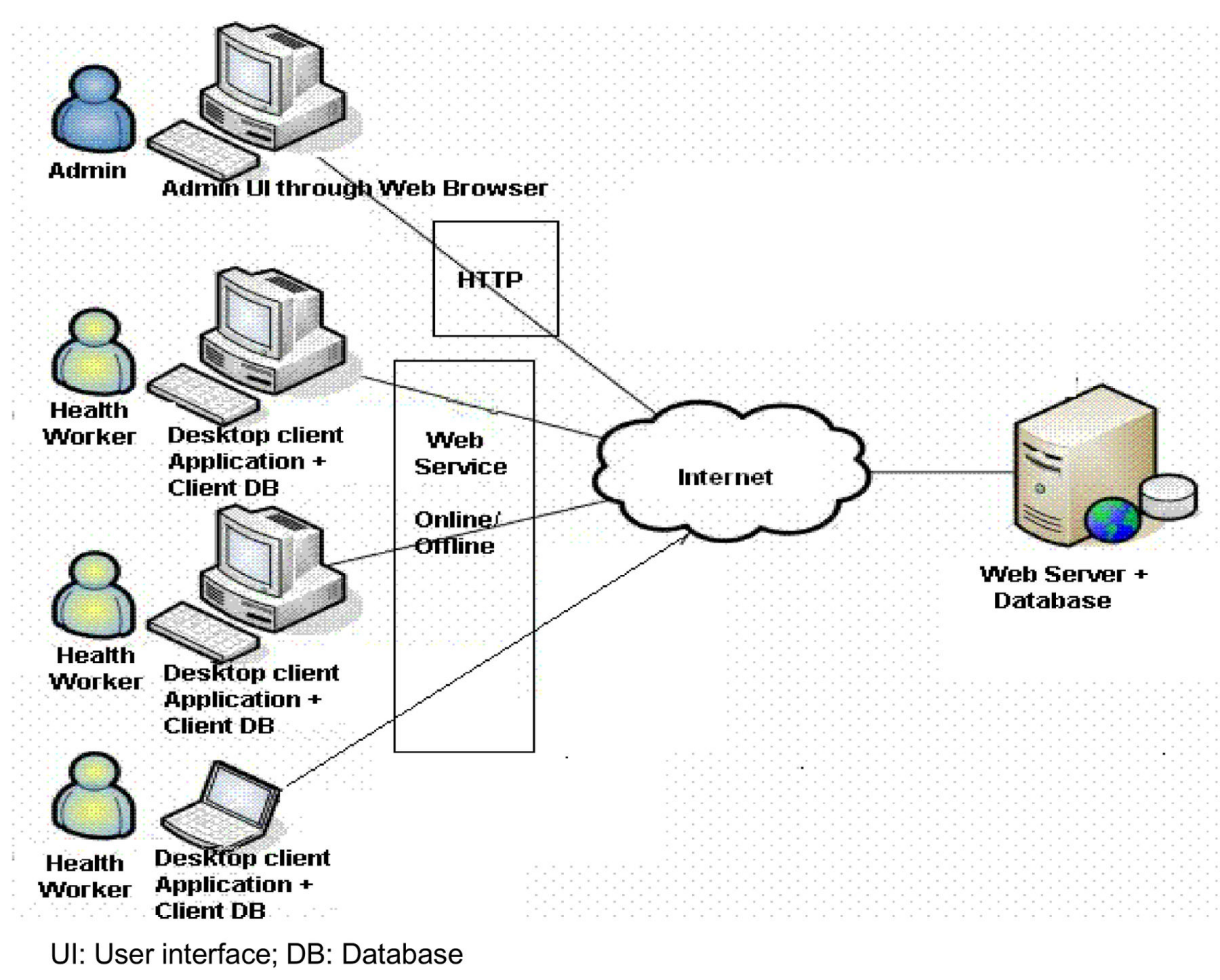
Architectural diagram for the DSS system.

Algorithms were developed by the software developers (Data Template, Bangalore, India) to help build the inferential engine base of the DSS. Medical language and software coding and machine language development were done by the medical software developers (Data Template) using "open source" platforms (JAVA and MySQL). Figure **2** details the architecture of the built DSS. Further details are mentioned in File S1. Caution was taken to ensure that the prepared ‘scenarios’, ‘risk stratifications’, ‘drug algorithms’ mirrored the Indian Hypertension II guidelines. The prepared “rules and logic” sheets [data collection form, drug indications, class of drugs and dosages, drug algorithms, undesirable combinations, risk stratification, referral scenarios and lab investigations (where possible)] were reviewed independently by two physician experts in the management of hypertension (government doctors from the non-participating PHC centers). 

### Phase II - Beta testing of DSS software

The contents of the user interface (UI) were shown to randomly selected physicians (from a line listed sample frame) who were working in the PHCs of Mahabubnagar district. The acceptability and validity of the questionnaire, reasoning for the questions, suggestions for improvement of data capture from the drop down menu, inputs on how to structure the summary page, views on what all the comprehensive elements need to be stressed in the tailor made recommendations, locally applicable and relevant life style advices that pop up in the DSS software upon entry of the data were field tested in 10% of physicians (n = 10) from the primary health care centers and from the community health care centers (n=8). The key feedback gained from the beta testing phase are summarised below:

#### (A) Clinical support required for management of hypertension

“Clear definitions of risk and staging of blood pressure, guidelines on effective lifestyle counseling in local language, advice on the best drugs from the available ones in the PHCs, information on the side effects, contraindications and benefits of each drug class among the antihypertensive medications, information buttons to cross check the recommendations and clear cut guidelines on when to refer a patient to the next level of care” were requested during beta testing phase

#### (B): Feasibility

Almost all the physicians and nurses felt that this was feasible provided it did not interrupt their daily workflow patterns. Key elements that were important to them were the accuracy of the recommendations, contents of the output, time taken for the input and the speed of the output from the system. Given the heavy workload of the outpatient departments in the PHCs, all agreed that a 10 minute window was the ceiling limit for the time taken between data entry and output of patient specific recommendations by the DSS.

#### (C) Operational issues

“Easily navigable, highly visible and understandable guidelines” were the main requests from all the participants. “Maintenance of the knowledge base and subsequent incorporation of new guidelines as and when they arose” were also requested. 

In addition to the life style counseling on benefits of losing weight and walking for at least 30 minutes a day; quitting smoking and avoiding alcohol; and adherence to medications, end users of the systems specifically requested for incorporation of locally applicable and relevant life style advices in the DSS. These included advice on harmful effects of locally prevalent forms of oral tobacco consumption (a) *khaini* - tobacco with slaked lime paste, and areca nuts (b) *zarda* - tobacco, lime, spices, vegetable dyes, areca nut (c) *pan masala* – betel leaf quid. Along with lifestyle advice to reduce addition of excess salt to prepared food, end users of the system also requested to incorporate counseling on reduction of papads (locally prepared highly salted snacks - seasoned dough made from lentils, chickpeas, rice, or potato, fried or cooked with dry heat) and pickles. Most users felt that figurative explanations for portion sizes for fruit and vegetable consumption would enable them to counsel better. Hence we defined portions as follows: A portion can be: vegetables (fresh, raw, tinned, or frozen) 1 portion = 3 tablespoons; salad, 1 portion = 1 bowl; fresh fruit, 1 portion = 1 medium apple, one banana; fruit juice (excluding cordials, fruit drinks, squashes), 1 portion = 1 small glass or more.

The time taken for completing the electronic data capture by the participating physicians was noted (mean time: 10 minutes, SD: 3 min), so as to achieve a consensus among them that it doesn’t affect their daily work flow patterns. This critical feedback from the field on the developed user interface was relayed to the developers. To understand the field level difficulties, paucity of the technical resources in a PHC and to gauge the OPD burden per day per center, visits were undertaken for a ‘passive observation’ along with the technical personnel. 

### Phase III - Validation of DSS in field settings

**Figure 3:**
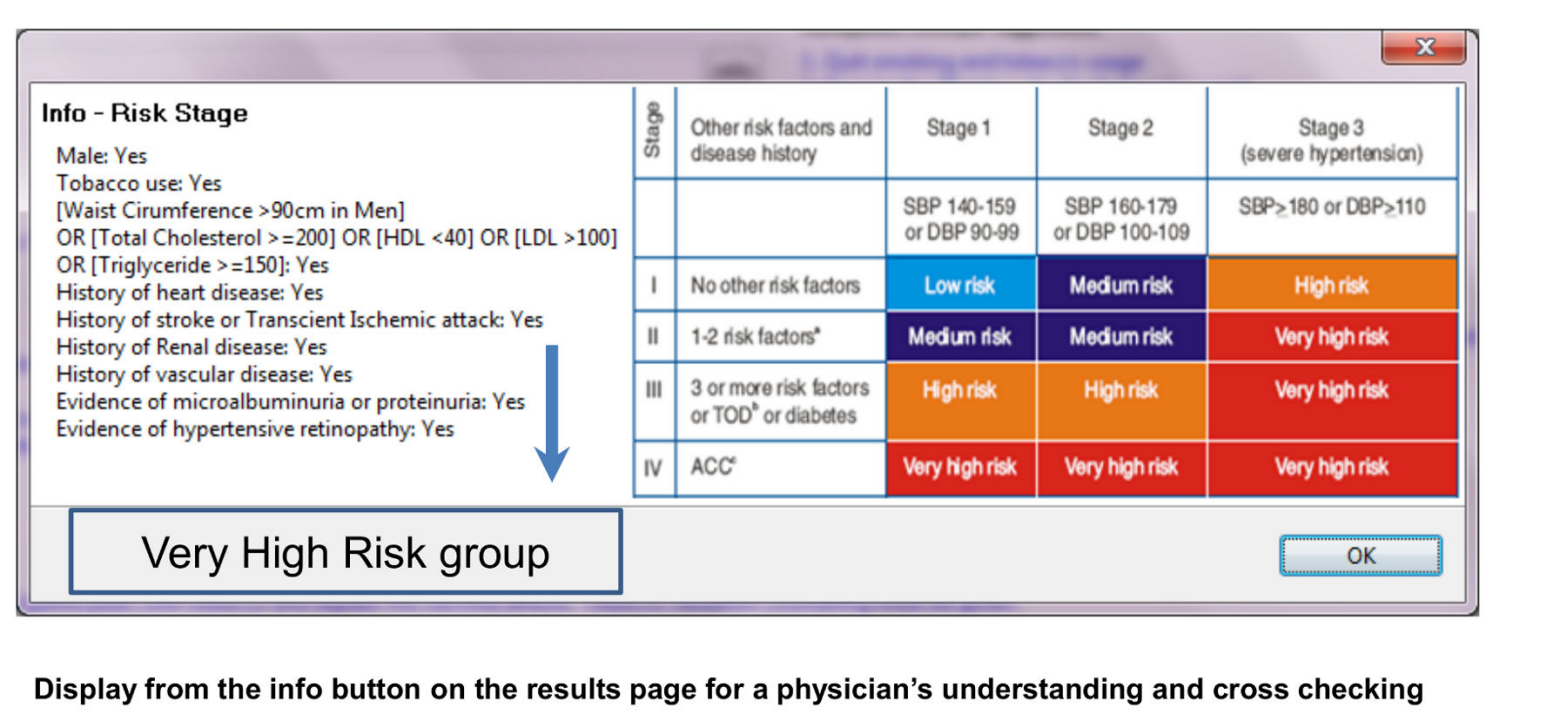
DSS screenshot showing “rules” and DSS engine logic based on 2007 Indian Hypertension II guidelines for risk staging.

**Figure 4:**
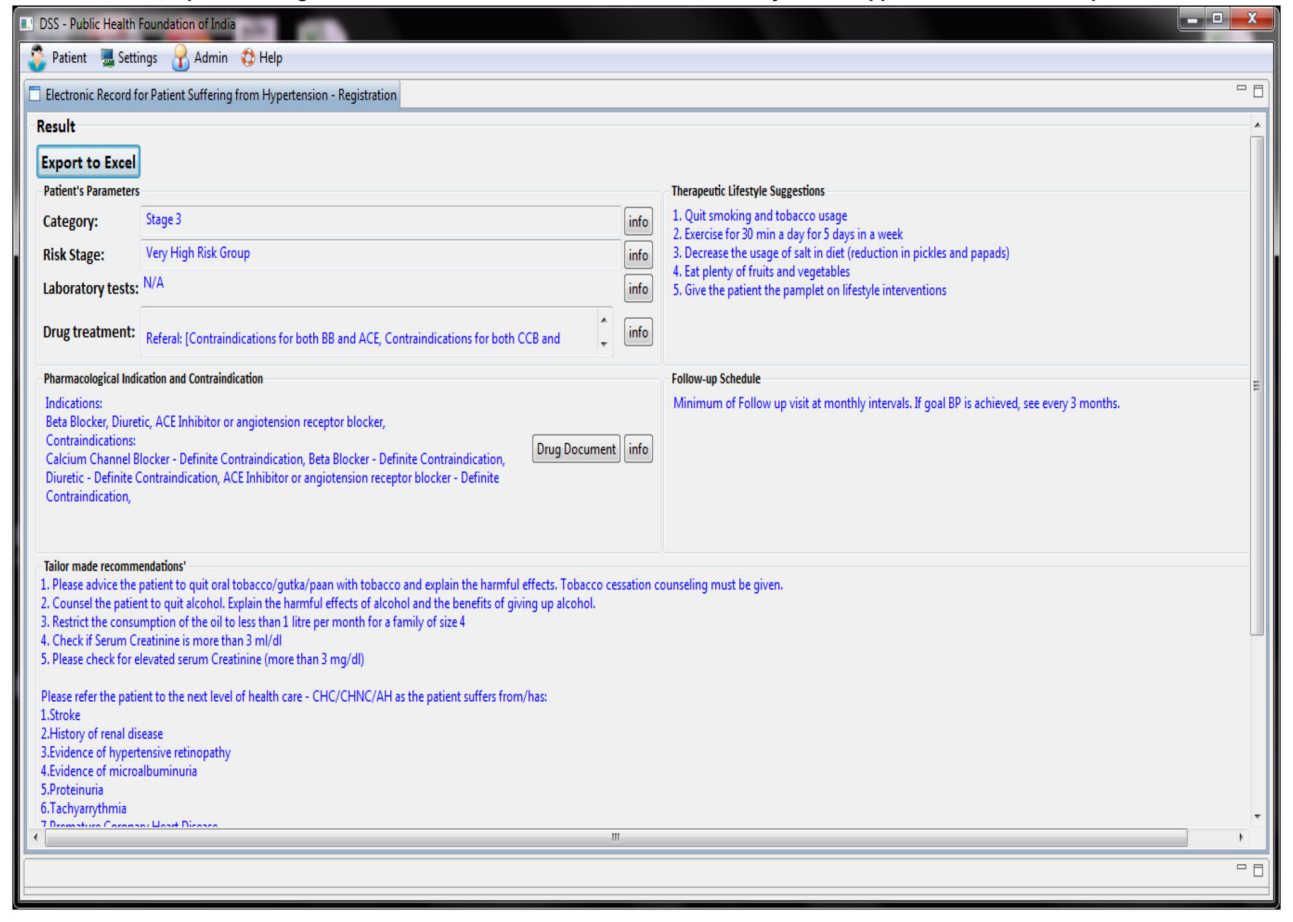
DSS screenshot showing staging of hypertension, risk category, and tailor made recommendations for drug treatment, pharmacological indication and contraindications, lifestyle support and follow up advice.

**Figure 5:**
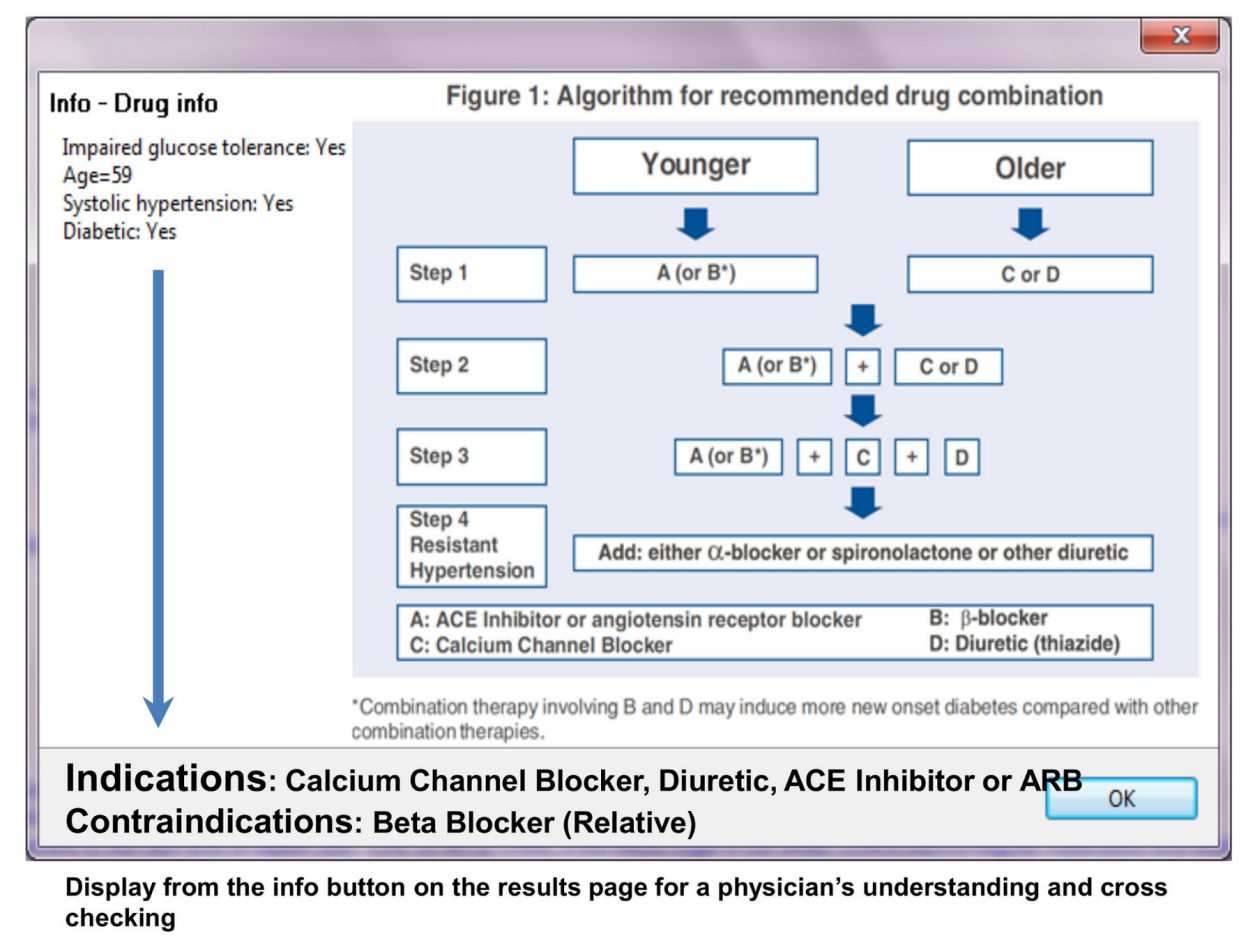
DSS screenshot showing the “rules” and DSS engine logic based on 2007 Indian Hypertension II guidelines for drug management.

We retrieved the systems risk staging (screen shot of DSS showing - Figure **3**), tailor made recommendations and the advice (screenshot of results page - Figure **4**) given to the patient (based on the clinical signs, symptoms and detailed history notes that the doctor entered in the netbook) from the field sites during the testing phase and compared them with the recommendations and advice given by two independent experts who were distinct from the two government doctors involved in phase one(experienced physicians from the government doctors from the non-participating PHC centers). The information and the reasoning logic displayed on ‘info’ buttons in the DSS output page (screenshot of info page- Figure **5**) was corroborated with the 2007 Indian hypertension guidelines. 

### Process and quality assurance

The quality process and the development of DSS software is summarised in table **1** and explained in detail in File S1. Testing was included in every iteration to ensure the quality of deliverables during the DSS development phase. The principal measure of progress was the delivery of ‘working’ software. Late changes in requirements were also welcomed as there was a close and daily cooperation between the business development people and developers of the DSS application. Face-to-face conversation (co-location), continuous attention to technical excellence and good design, simple self – organising teams with adaptability to changing circumstances were the key quality assurance norms followed during the DSS development, beta testing, field testing, pilot testing and finally during the implementation phase. 

**Table 1:** Development process for the DSS software.

**Units**	**Activities**
Unit I	Detailed system analysis; technology evaluation, design documentation and user interface (UI) design; algorithm development; creation of login/logout, doctor and location details; security feature implementation; and phase release for testing
Unit II	Patient registration, patient's consequent visit details entry, and development of algorithms for consequent visit with comparison
Unit III	Conversion to PDF of the user details; UI for the advice with print support; synchronization option with the centralized database (DB); version checking and update of patient data; alerts and message prompting; scheduler for DB backup; installer for the application; and phase release for quality audits (QA) and production
Unit IV	Installation Guide; user guide and user acceptance testing (UAT)

### Statistical Methods

Sample size: The number of subjects required in a 2-rater study to detect a statistically significant (p<.05) on a dichotomous variable, with 80% power, at various proportions of positive diagnoses, to detect a difference in kappa of 0.40 is 50 for a two tailed test [15]. Assuming a response rate of 80%, the final sample size was adjusted to 60 Indian hypertensive patients. The detailed calculations for the overall agreement, kappa and 95% CI values are mentioned in File S1.

## Results

The overall percent agreement (calculated as the sum of all agreements divided by the total number of observations) between the DSS software and the independent experts (not from the study team) on the stage of the blood pressure (BP) measurement, risk, drug management, side effects and adverse interactions, lifestyle advice and follow up advice was 90% (95% CI: 88.52 - 90.16); 91.67% (95% CI: 90.16 - 91.80); 85% (95% CI: 83.61 - 85.25); 86.67% (95% CI: 85.25 - 86.89); 90% (95% CI: 88.52 - 90.16); and 83.33% (95% CI: 81.97 - 83.61) respectively (table **2** ). Among 60 hypertensives, the decision making of the DSS versus the opinion of the independent experts (distinct from the two government doctors involved in phase one) matched for staging of the BP in 54 HTN patients, risk stratification done by the DSS matched the expert’s opinion in 55 HTN patients, drug management algorithm suggested by the DSS matched that of the expert in 51 HTN patients. The recommendations suggested for lifestyle advice, follow up advice and adverse interactions matched in 54, 50 and 52 HTN patients respectively. 

**Table 2:** Percent agreement, lower and upper bounds for attributes between DSS and independent expert.

**Attribute**	**m**	**n**	**v1**	**v2**	**u**	**v3**	**v4**	**w**	**% agreement**	**LB %**	**UB%**
Stage of BP	54	60	108	14	2.56	110	12	0.48	90.00	88.52	90.16
Risk category	55	60	110	12	2.79	112	10	0.46	91.67	90.16	91.80
Drug management	51	60	102	20	2.39	108	14	0.50	85.00	83.61	85.25
Life style advice	54	60	108	14	2.56	110	12	0.48	90.00	88.52	90.16
Follow up advice	50	60	100	22	2.09	102	20	0.54	83.33	81.97	83.61
Adverse event	52	60	104	18	2.27	106	16	0.52	86.67	85.25	86.89

m = number of observations where DSS and expert agreed; n = total number of observations; v1 = 2*m; v2 = 2*(n - m + 1); u = the 2.5th percentile of the F distribution with v1 and v2 degrees of freedom; v3 = 2*(m+1); v4 = 2*(n-m); w = 97.5th percentile of the F distribution with v3 and v4 degrees of freedom; % agreement = (total number of agreements/total sample size); LB% = lower bound of confidence interval in percentage; UB% = upper bound of confidence interval in percentage

**Figure 6:**
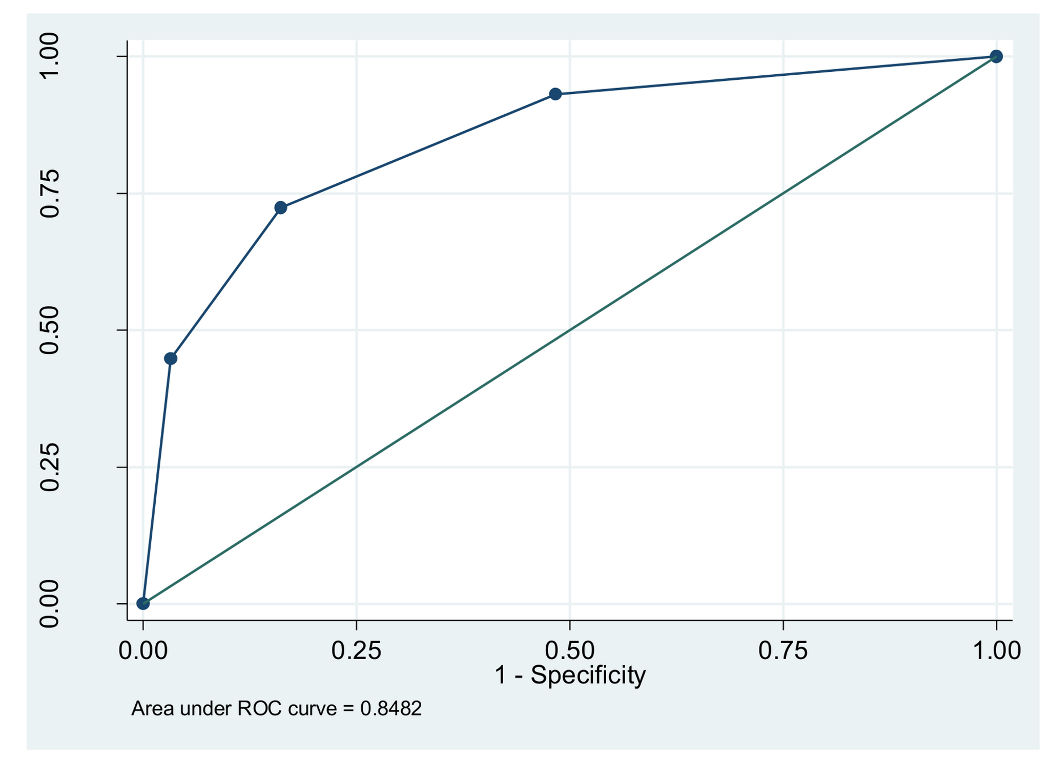
Receiver Operator Curve for comparing the DSS and independent experts on drug management.

Based on the risk category; staging of BP; presence or absence of associated clinical conditions and target organ damage; DSS suggested drug management for 39 out of 60 HTN patients, whereas the independent experts opined that 42 out of 60 would need drug management (table **3** and table S1 in File S1). The positive and negative percent agreements were 85.71% and 83.33 % respectively. The overall percent agreement (P_o_) between the DSS and expert was 85% (95% CI: 83.61% - 85.25%). Receiver Operator Curve (ROC) showed a good accuracy for the DSS, wherein, the area under curve (AUC) was 0.848 (95% CI: 0.741 - 0.948). Sensitivity and specificity of the DSS were 83.33 and 85.71% respectively when compared with independent experts (Figure **6**). The kappa statistic (observed agreement beyond chance divided by the maximum agreement beyond chance), for overall agreement for drug management was 0.659 (95% CI: 0.457 - 0.862) indicating a substantial degree of agreement beyond chance at an alpha fixed at 0.05 with 80% power. The prevalence index was 0.31 and the bias index (extent to which the raters disagree on the proportion of positive or negative cases) was 0.05. 

**Table 3:** 2*2 cell depicting the real versus virtual for drug management in HTN.

DSS - suggestions	Independent expert suggested drug treatment	Independent expert did not suggest drug treatment
DSS suggested drug treatment	36 (a)	3 (b)
DSS did not suggest drug treatment	6 (c)	15 (d)

## Discussion

We built a clinical decision support system, based on 2007 Indian hypertension II guidelines, for staging and risk stratification of hypertension, for suggesting evidence based recommendations on drug management and life style advice of hypertensive patients to better manage hypertensive patients at a primary healthcare level. We report a good accuracy of our built DSS, with an AUC of 0.848 (95% CI: 0.74-0.94) with sensitivity and specificity values of 0.83 and 0.85 respectively. A moderate to substantial agreement of 0.66 (estimate for kappa) on drug management of hypertensive patients was noticed between the DSS and an independent expert after adjusting for occurrence of a chance agreement. The 95% CI for kappa fell in the fair to substantial agreement range (0.43 to 0.89). 31% of agreements on the positive classification differed from that of the negative classification and the disagreement between the DSS (virtual) and the independent expert (real) on the proportion of positive or negative cases was 5%. The prevalence and bias index, the 95% CI for kappa and positive and negative agreements of 0.91 and 0.73 indicate the robustness of the yielded kappa. 

### Comparison with previous literature

One of the first specific DSS built for managing hypertension, the ATHENA-Hypertension (Assessment and Treatment of Hypertension: Evidence-based Automation built by Stanford Medical Informatics) system [13], a similar knowledge-based DSS like our built DSS, showed that implementation and deployment of clinical decision support was feasible in large clinical settings [14]. Differences in ATHENA system and DSS in drug management, prescription of antihypertensives, availability of physiological testing and risk classification are explained in detail in the File S1. The clinical data visualizations and evidence to support specific recommendations in ATHENA were more comprehensive than the physician in adding, substituting or increasing drug therapy, where the criteria were clear in the pre-defined rules [13,15]. Our study showed a moderate to substantial agreement on drug management of hypertensive patients between DSS and the independent physician evaluators. Care was taken to ensure that testing data came from real patients and representative physician evaluators who were familiar with the clinical settings similar to the offline ATHENA system testing study (a ‘physician-evaluator who was a representative of the end-user population’ validated the system) [15]. 

The concordance rates for definite indication and absolute contraindication for drug management in hypertension were 85% and 100%, respectively when the knowledge base for a hypertension management DSS (LIGHT) was verified and validated [18]. We report similar findings from our study, i.e., positive and negative percent agreements were 85.71% and 83.33 % respectively and the overall percent agreement (P_o_) between the DSS and experts was 85% (95% CI: 83.61% - 85.25%). In an on-demand DSS study for primary care management of hypertension (similar clinical settings of primary health care in our study), physicians were more willing to use DSS in complex clinical situations, when the reasoning logic was clearly demarcated [19]. Our DSS has display buttons for information on the logic and engine rules that help in arriving at decisions based on patient profile and clinical history. 

A recent paper on ‘Analysis on the accuracy of a decision support system for hypertension monitoring’ on a developed DSS – the WeHealth system [20], proposed a theoretical method to evaluate the accuracy of WeHealth hypertension monitoring system by linking the system accuracy with the distribution of sensors’ errors (systolic and diastolic BP) and the errors of context (entry risk factors, target organ damage and complications). The difference in accuracy was less than 1% when traditional (physician review) was compared with the WeHealth DSS [20]. The difference in accuracy between our DSS and the independent experts was in between 2-4% (table S2 in File S1). Moreover the value of AUC of 0.848 with a tight range of 95% CI (0.74-0.94) suggests a good accuracy of our DSS.

### Strengths

Our DSS has user friendly and properly structured (recommendation and reasoning info buttons) pull-down lists; a consistent use of information or use of symbols and color for improving visibility and speed of navigation; a clinical user interface that mimics their paper predecessors; and has a standardized evidence based risk stratification, staging of BP, guidelines and recommendations for drug, lifestyle and follow up advice for Indian patients suffering from hypertension. The decision algorithm is also visible as a pop up menu for the clinicians to see and find out the logic behind the decision. Moreover, we have taken into confidence and involved the end users of the DSS (clinicians and treating physicians at a primary health care level) at every stage of the development, pilot testing and validations so that a consistent understanding of the purpose of the DSS system and the functionality of the user interface takes place during the implementation phase. Incomplete or inaccurate data entry has been prevented as the ceiling (maximum and minimum permissible values) limits for each variable have been defined during the coding process. A summary sheet highlighting the patient specific key risk factors, stage of BP, any co-morbid conditions is in place, so that the cognitive burden of absorbing the information does not prevent the end users from thinking about what the information means.  We have validated the DSS by attempting to simulate a real life scenario by bringing in independent evaluators who were not otherwise involved in the DSS project, but were a part of the government run primary health care system care givers.

The SAGE (Standards-Based Sharable Active Guideline Environment) consortium project recommends that a DSS should have “a complex clinical guideline as a series of recommendation sets” [21]. Our DSS takes into account the context, decision, action and route to create a standards-based decision support’s system. We followed the SAGE guidelines model which suggests that DSS (a) must be delivered through features available within the existing clinical information systems (b) must facilitate clinical workflow non-intrusively and (c) must be efficient and allow easy inspection of the underlying clinical logic [22]. The fundamental principle involved in evaluating methods in medical informatics is to do a comprehensive evaluation of the consistency, depth and coverage of the knowledge encoded in the system [23]. Each of these areas was tested in our DSS validation. Finally, implementation of clinical guidelines through the DSS acts as a teaching tool for the treating physicians and also ensures adherence to current guidelines resulting in quality of health care services provided.

Miller et al [24] underlined the importance of thinking through the necessary key features during the process of developing a medical diagnostic and treatment algorithm. The validation for the system performance was based on what clinical practitioners would use or require during actual practice. The boundaries and limitations of the knowledge-base and available system functions have been specified upfront. Particular emphasis was paid to address the system-related (unambiguous and easy navigability of end user interface), user-related (lack of training with the system, failure to understand key system functions, lack of medical knowledge, etc.), and external variables (lack of available gold standards, quality of independent reviewers) influences on the validation process.

### Limitations

Developing a standard for comparing the DSS recommendations turned out to be a challenge. Although, physician reviews have traditionally served as the gold standard, errors owing to the large and voluminous data analysis (60 patients’ history and physical findings) may have limited the validity. Similarly, the authors of ATHENA-DSS study also acknowledge that an evolved consensus between the physician review and recommendations put forth by the system could turn out to be a better gold standard [15]. In the CHAID (Chi-squared Automatic Interaction Detection) DSS for hypertension management built by using a data mining approach, clustering and the association rules were used for validating decisions made in hypertension management [25]. More specifically, data warehouse architecture was used to collect and integrate relevant data from hospital clinical information systems. Our study, being a pilot study to test out the feasibility of converting clinical practise guidelines into implementable and hands on decision rules doesn’t integrate all the data from patient electronic records and hospital clinical information systems as the infrastructure for health management information system is still at a nascent stage in India.

The knowledge based engine in our system, built mostly on “if and then” scenarios limits itself to management of hypertension only at primary care settings. Similarly, potential interactions with other drugs that would have had an effect on blood pressure have not been built in the system. Referral scenarios are suggested when the reasoning engine is confronted with complex data that can be managed only at secondary and tertiary care settings. However, since the issue of random agreement purely by chance has been adequately addressed, we believe that our finding of moderate to substantial agreement between the virtual (DSS) and the real (physicians review) are valid (since we report the AUC and the 95% CI for AUC in the ROC curve, 95% CI for kappa, prevalence and bias index).

DSS used in the developed world for management of hypertension have shown success if the DSS seamlessly blends in the daily work patterns of the end users, without burdening them on the cognitive or time scales, and improves their work efficiency. The time spent on manual data entry, loss of opportunity for decision, and the onus or responsibility in the event of an error are major areas that need to be addressed in future DSS studies. We have followed a systematic approach for DSS validation study wherein, feasibility, reliability in performance, DSS components’ testing, evaluation of DSS in the context in which they were developed were initially done before a randomised control trial was planned. The results of the just completed randomised trial [26] will help us to undertake a formal evaluation of DSS on patient specific outcomes. 

## Conclusion

A point of care, pilot tested and validated virtual DSS which matches the real life scenario for management of hypertension has been developed for improved management of hypertension at a primary health care level in a low and middle income setting. Public health policy decision makers could use the innovative DSS platform for (a) delivering evidence based non communicable disease (NCD) health care delivery models (promotion, prevention and treatment), (b) improving health system efficiency, and (c) reducing health disparities in primary care settings in low and middle income (LMICs) countries. 

## Supporting Information

File S1 Supplementary data file.
(DOCX)
