# Supplementary material for: Development and Validation of a Clinical and Computerised Decision Support System for Management of Hypertension (DSS-HTN) at a Primary Health Care (PHC) Setting
Source: PLoS One. 2013 Nov 5;8(11):e79638. doi: 10.1371/journal.pone.0079638 (PMC3818237; doi:10.1371/journal.pone.0079638)
Supplement: File S1 — Supplementary data file. (DOCX) [file pone.0079638.s001.docx]

**File S1 (Supplementary data file)**

**Expanded Results and discussion**

**Table S1: 2*2 cell depicting the real versus virtual for drug management in HTN**

| DSS - suggestions | Independent expert  suggested drug  treatment | Independent expert  did not suggest drug  treatment |
| --- | --- | --- |
| DSS suggested drug treatment | 36 (a) | 3 (b) |
| DSS did not suggest drug treatment | 6 (c) | 15 (d) |

Overall percent agreement (P_o_) between the DSS and experts for drug management was

85 % (95% CI: 83-88)

**(A) Calculations for P_o_ ( Overall percent agreement) and 95% confidence limits for drug management:**

P_o_ = Overall percent agreement

m = the number of cases where all appraisers’ assessments agree with each other

n = the total number of samples inspected

P_o_ = 51/60 = 0.85

Calculations for 95% Lower Bound (alpha = 0.05) for Po

v1 = 2 * m = 2*51 = 102

v2 = 2*(n - m + 1) = 2(60-51+1) = 20

u = F(v1, v2, 0.025) = the 2.5th percentile of the F distribution with v1 and v2 degrees of freedom

u= F(102,20,0.025) = 2.17

Lower bound = (v1 * u) / (v2 + v1 * u) = 221.24/ 264.74= 0.83

Calculations for 95% Upper Bound (alpha = 0.05) for Po

v3 = 2*(m+1) = 2*52 = 104

v4 = 2*(n-m) = 2*9 = 18

w = F(v3, v4, 0.975) = the 97.5th percentile of the F distribution with v3 and v4 degrees of freedom

F(104,18,0.975) = 0.53

Upper bound = (v3 * w) / (v4 + v3 * w) = (104*0.53) / (122*0.53) = 55.12/64.66 = 0.85

**(B) Calculations for P_o_ ( Overall percent agreement) and 95% confidence limits for staging of BP:**

P_o_ = 54/60 = 0.90 ; (95% CI: 0.88 – 0.91)

95% Lower Bound (alpha = 0.05)

v1 = 2 * m = 2*54 = 108

v2 = 2*(n - m + 1) = 2(60-54+1) = 14

u = F(v1, v2, 0.025) = the 2.5th percentile of the F distribution with v1 and v2 degrees of freedom

u= F(108,14,0.025) = 2.56

Lower bound = (v1 * u) / (v2 + v1 * u) = 276.48/ 312.32= 0.88

95% Upper Bound (alpha = 0.05)

v3 = 2*(m+1) = 2*55 = 110

v4 = 2*(n-m) = 2*6 = 12

w = F(v3, v4, 0.975) = the 97.5th percentile of the F distribution with v3 and v4 degrees of freedom

F(110,12,0.975) = 0.48

Upper bound = (v3 * w) / (v4 + v3 * w) = (110*0.48) / (122*0.48) = 52.8/58.56 = 0.91

**(C ) Calculations for PPA and NPA**

The overall percent agreement can be calculated as (a+d)/(a+b+c+d)

Positive percent agreement (PPA) = a/(a+c) = 36/42 = 0.85 ;

Negative percent agreement (NPA) = d/(b+d) = 15/18 = 0.83

**(D) Calculations for Accuracy for Risk categorization among hypertensive patients**

Accuracy (ACC) = probability of (decision in low risk, truth in low risk) +pr (decision in medium risk, truth in medium risk) + pr(decision in high risk, truth in high risk) +pr (decision in very high risk, truth in very high risk)

Reference for the above formulae: John Robert Taylor (1999). An Introduction to Error Analysis: The Study of Uncertainties in Physical Measurements. Second edition. University Science Books. 128C129.

**Table S2: 2*2 cell depicting the real versus virtual for risk staging**

|  | Probability of correct staging by DSS | Probability of correct staging by Independent experts |
| --- | --- | --- |
| Low risk | 12/12 = 1 | 12/12 = 1 |
| Medium risk | 22/23 = 0.95 | 21/23 = 0.91 |
| High risk | 17/19 = 0.89 | 17/19 = 0.89 |
| Very high risk | 6/6 = 1 | 5/6 = 0.8 |

DSS = (3.84/4) = 0.96

Independent experts = (3.63/4) = 0.91

Difference in accuracy of DSS and real context = 4%

Difference in accuracy of independent experts and real context = 9%

**(E) ROC curve values from STATA**

**(F) Differences in ATHENA system and our decision support system (DSS)**

*Drug management*

The ATHENA system suggests life style modification for up to 12 months for risk group A who suffer from Stage 1 blood pressure (BP), whereas our DSS based on Indian Hypertension 2007 guidelines suggest lifestyle modification for up to 3 months. If BP is not under control and the end of three months of lifestyle modification, the system suggests initiation of drug management.

The ATHENA system suggests life style modification for up to 6 months for risk group B who suffer from Stage 1 blood pressure (BP), whereas our DSS based on Indian Hypertension 2007 guidelines suggest lifestyle modification for up to 3 months. If BP is not under control and the end of three months of lifestyle modification, the system suggests initiation of drug management.

*Available antihypertensives*

Only Hydrochlorothiazide (6.25-25 mg dosage), Atenolol (25-100 mg dosage), Amlodipine (2.5-10 mg dosage), Enalapril (2.5-5 mg dosage) are available in the study centres (primary health care centres in Mahabubnagar district, AP, India). Angiotensin receptor blockers (Losartan) are not available at the primary care level in the government sector.

*Physiological tests*

Facilities for testing for lipids (LDL, HDL, and triglycerides), proteinuria (albuminuria and creatinine clearance tests) and electrocardiogram (ECG) or Echocardiography are not available in the primary health care (study centres). They are only available at the next level of care – community health centres.

*Risk factor classification*

The originally developed ATHENA system was developed based on The Sixth Report of the Joint National Committee on Prevention, Detection, Evaluation, and Treatment of High Blood Pressure, had classified the risk into (a) Risk Group A – absence of any risk factors (smoking, dyslipidaemia, diabetes mellitus, age more than 60 years for males or post-menopausal female, family history of MI in men< 55 or women < 65 years), no target organ damage (TOD) or clinical cardiovascular disease (CCD); (b) Risk Group B – presence of at least one risk, no DM, no TOD or CCD; and (c) Risk Group C-presence of diabetes mellitus and/or TOD / CCD.

Our decision support system for management of hypertension is based on Indian Hypertension 2007 guidelines which have classified risk as low, medium, high and very high risk:

(a) Low risk: Stage 1 BP with no risk factors; (b) Medium risk: Stage 1 BP with 1-2 risk factors; stage 2 BP with no risk factors or stage 2 with 1-2 risk factors; (c)High risk: Stage 1 BP with 3 or more risk factors or diabetes mellitus or presence of target organ damage; Stage 2 BP with 3 or more risk factors or diabetes mellitus or presence of target organ damage; Stage 3 BP with no risk factors; and (d) Very high risk: Stage 1 or 2 or 3 with presence of CCD; Stage 3 BP with 3 or more risk factors or diabetes mellitus or presence of target organ damage

**(G) Software development process and quality assurance**

DSS application was created using client server architecture wherein information exchange can take place between a server and multiple clients. Client was designed to gather data from users and server was responsible for storing the data in a single database. A high level architectural diagram for the system is depicted in figure 2.

The client application was built to run on computers, laptops and notebooks. Client could work in both offline and online mode. When on online, the server was contacted for various data related operations.

Components of DSS:

- 1. Desktop Client Application
  2. Server Application

1. *Desktop Client Application*

Desktop Client Application was designed for Health workers to capture the patient’s information and measurements and to execute the algorithm. This application was developed in Java Programming language. Along with java, Standard Widget Toolkit (SWT) and Eclipse Rich Client Platform were used to build the application. To store data at the client side, this application uses Structured Query Language (MySQL) database.

1. *Server Application*

Consolidated data from the client applications running at various machines will be stored in a centralized server machine. This machine ran a server application which was capable of collecting the data from the client applications and to do reporting. This application was deployed in a Glassfish application server software. To store data, it used a MySQL database. The server application also had an admin module part and the same was accessible via a web application using http protocol.

Client application was able to communicate to the server using web-service technology.

Software platforms utilised for DSS development

(a) *Java Platform, Enterprise Edition or Java EE:* DSS was built using java based open source and freeware technologies. This helped in cutting down the total development cost to a large extent. Java is one of the most commonly used programming languages in the software development world. Some key advantages of java when compared to the other technologies, such as, wide applicability of java from computers to mobile devices, ability of java to run independently on any platform (windows, linux and unix), ability of java to run on various environments (web, application server) were the reasons for utilising java in the development phase.

(b) *MySQL* - is the world's most used relational database management system (RDBMS) that runs as a server providing multi-user access to a number of databases. MySQL development project has made its source code available under the terms of the GNU General Public License, as well as under a variety of proprietary agreements.

(c) *Glass Fish server:* is an open-source application server project started by Sun Microsystems for the Java platform, and now sponsored by Oracle Corporation. This is purely a java based server and is again a freeware.

(d) *Spring Framework* *and Hibernate*: We used spring framework as open source server side application frame work. **Hibernate** enables the application to access data from any database in a platform-independent manner. All the necessary details for accessing a particular data source are easily configurable in Xml files.

(e) *Standard Widget Toolkit (SWT)*: is a graphical widget toolkit, and one of the most commonly used windows developer build using java. SWAT was chosen as it is faster and gives a much better look and feel for the application.

(f) *Web service:* is the most commonly used platform independent communication mechanism in a client server environment. We used Jersey for the implementation.
